# The insulin like growth factor and binding protein family: Novel therapeutic targets in obesity & diabetes

**DOI:** 10.1016/j.molmet.2018.10.008

**Published:** 2018-10-24

**Authors:** Natalie J. Haywood, Thomas A. Slater, Connor J. Matthews, Stephen B. Wheatcroft

**Affiliations:** Division of Cardiovascular and Diabetes Research, Leeds Multidisciplinary Cardiovascular Research Centre, Faculty of Medicine and Health, University of Leeds, United Kingdom

**Keywords:** Insulin like growth factor, Obesity, Diabetes, Insulin like growth factor bind protein

## Abstract

**Background:**

Recent changes in nutrition and lifestyle have provoked an unprecedented increase in the prevalence of obesity and metabolic disorders. Recognition of the adverse effects on health has prompted intense efforts to understand the molecular determinants of insulin sensitivity and dysglycemia. In many respects, actions of insulin-like growth factors (IGFs) mirror those of insulin in metabolic regulation. Unlike insulin, however, the bioactivity of IGFs is regulated by a family of seven high-affinity binding proteins (IGFBPs) which confer temporospatial modulation with implications for metabolic homeostasis. In addition, evidence is accumulating that IGF-independent actions of certain of the IGFBPs can directly modulate insulin sensitivity.

**Scope of review:**

In this review, we discuss the experimental data indicating a critical role for IGF/IGFBP axis in metabolic regulation. We highlight key discoveries through which IGFBPs have emerged as biomarkers or putative therapeutic targets in obesity and diabetes.

**Major conclusions:**

Growing evidence suggests that several components of the IGF-IGFBP system could be explored for therapeutic potential in metabolic disorders. Both IGFBP-1 and IGFBP-2 have been favorably linked with insulin sensitivity in humans and preclinical data implicate direct involvement in the molecular regulation of insulin signaling and adiposity respectively. Further studies are warranted to evaluate clinical translation of these findings.

## 1 Introduction

Worldwide the proportion of adults with a body mass index (BMI) of 25 kg/m^2^ or greater is 36.9% in men and 38% in women. Worryingly, prevalence has increased substantially in children and adolescents in developed countries over the last few decades; 23.8% of boys and 22.6% of girls were overweight or obese in 2013. This trend is not confined to developed countries [1]. Obesity associated complications, including cancer, type 2 diabetes mellitus (T2DM) and cardiovascular disease, are becoming an increasing burden to healthcare systems worldwide. It is predicted that type 2 diabetes will affect 300 million people globally by 2030 [2]. T2DM is a major cause of atherosclerosis, leading to premature myocardial infarction, stroke, and peripheral arterial disease; in both men and women, diabetes confers an equivalent degree of risk as aging about 15 years [3]. Although intensive lowering of blood glucose is now achievable in most individuals with diabetes, this strategy does not effectively reduce macrovascular disease events [4], [5], and novel therapeutic targets need to be identified.

The hormone insulin and related peptides insulin-like growth factors (IGFs) have diverse actions in mammalian physiology. Their crucial role in growth and development is well understood. Although insulin plays a fundamental role in the maintenance of normal blood glucose levels, it is accepted that IGF-I exerts complementary effects in glucose counter-regulation. Recently, important roles of other members of the IGF axis, particularly the IGF binding proteins, have become apparent in obesity and diabetes, and could potentially be exploited therapeutically.

## 2 IGF/binding protein axis

**Figure 1:**
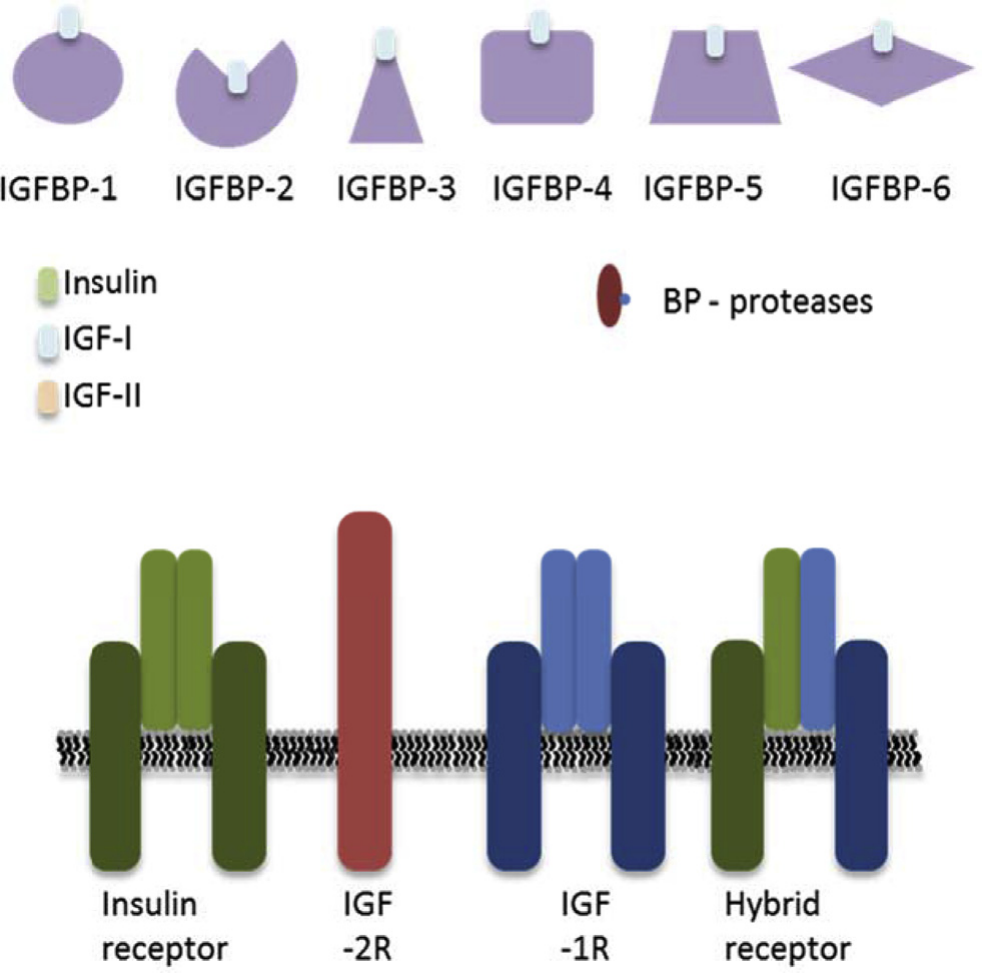
**The IGF/insulin axis.** There are 4 types of receptor within the IGF axis; the insulin receptor, the IGF-1R, the IGF-2R and the hybrid receptor. There are 3 ligands, Insulin, IGF-I, and IGF-II that bind to the 4 receptors, with varying affinities. The insulin receptor exists as two isoforms IR-A and IR-B and IGF-1R can hybridize with both forms. Finally, within the axis there is a family of 6 IGF-I binding proteins named IGFBP-1-6 and several families of binding protein proteases that regulate the binding proteins.

IGF-I and -II are evolutionarily conserved peptide hormones with structural homology to proinsulin [6]. There are two known IGF receptors - the type 1 IGF receptor (IGF-1R) and the IGF-II receptor (IGF-2R). Six IGF binding proteins have been identified (IGFBP-1-6). BP-related protein (IGFBP-rP1, also known as IGFBP-7) also contributes to the ‘superfamily’ of proteins that bind the IGFs (Figure 1). IGFBPs act as transport proteins, prolong half-life of IGFs, regulate the clearance of the IGFs, provide a tissue specific localization, and directly modulate IGFs' actions [7]. It is now also accepted that the majority of the IGFBP have IGF independent actions.

The primary structure of mammalian IGFBPs contains three distinct domains of roughly equivalent sizes. The N-terminal and C-terminal portions are highly conserved across the IGFBP family and are responsible for IGF binding. The central linker domain is the least conserved region, comprising sites affected by post-translational modifications including proteolysis, glycosylation, and phosphorylation. Some IGFBPs possess distinctive structural motifs such as an integrin recognition sequence and heparin binding motifs. These distinctive characteristics, highlighted in Table 1, may contribute to the IGF independent actions of the binding proteins [7].

**Table 1:** Characteristics of individual IGFBPs.

Binding protein	C-terminus	Central linker domain	N-terminus
IGFBP-1	• IGF binding	• Phosphorylation sites	• IGF binding
	• Integrin binding		
IGFBP-2	• IGF binding	• Heparin binding	
	• Integrin binding		
IGFBP-3	• IGF binding	• Heparin binding • Phosphorylation sites • N-glycosylation	• IGF binding • Insulin binding • Inhibitor of IR auto-phosphorylation • Inhibitor of mitogenesis
	• Cell binding/penetration (GAG domain)		
	• Nuclear localization signal		
	• Heparin binding		
	• ALS (Acid labile subunit) binding		
IGFBP-4	• IGF binding	• N-glycosylation	• IGF binding
IGFBP-5	• IGF binding	• ALS binding • Heparin binding • Phosphorylation sites	• IGF binding
	• Nuclear localization signal		
	• Heparin binding		
	• ALS binding		
IGFBP-6	• Heparin binding	• N-glycosylation	• IGF binding
	• IGF-II binding		
IGFBP-7		• Kazal-like domain • N-glycosylation	• Ig-like C2-type

### 2.1 IGF-I

**Figure 2:**
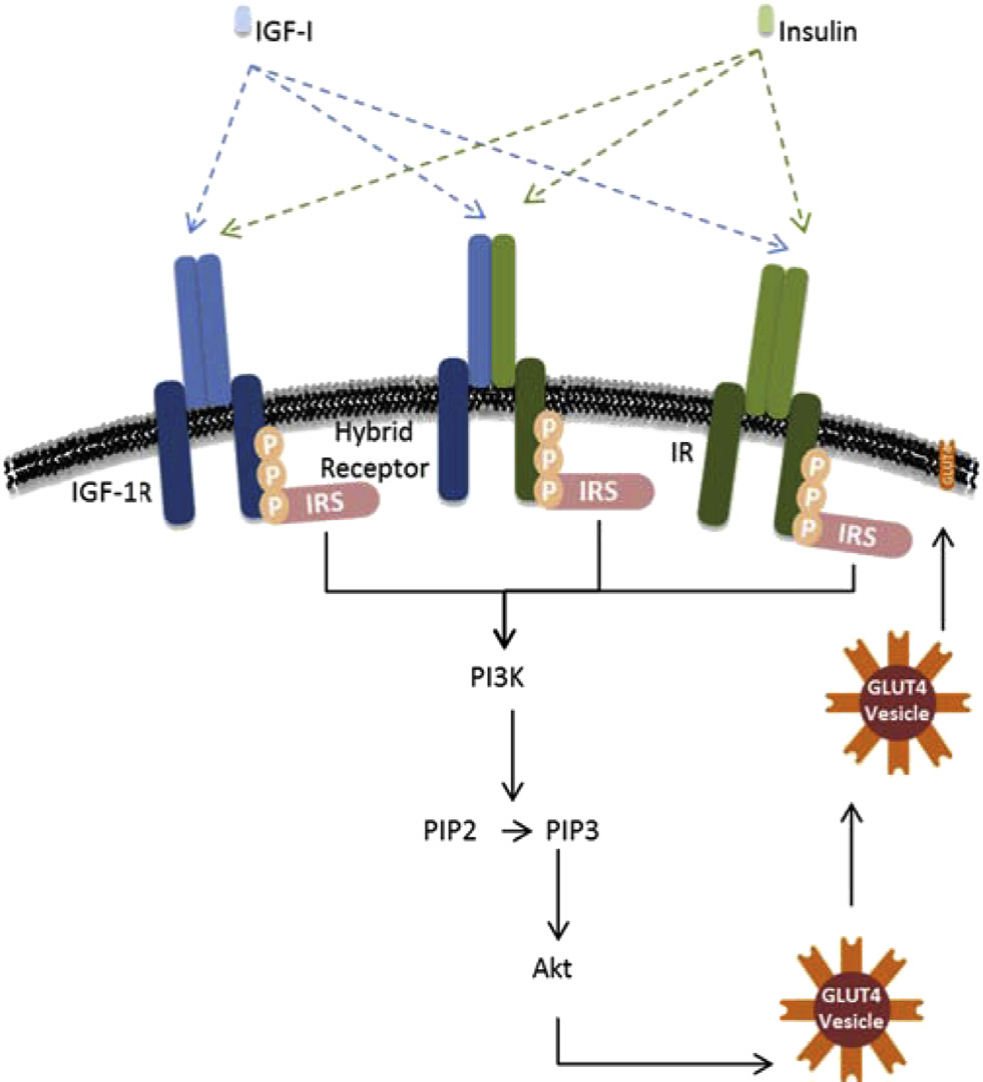
**– IGF-I/Insulin PI3K/AKT signaling pathway.** Insulin elicits a diverse array of biological responses by binding to its specific receptor. The insulin receptor (IR) belongs to a subfamily of receptor tyrosine kinases that includes the IGF type 1 receptor (IGF-1R). These two receptors can form hybrids. These receptors are tetrameric proteins consisting of two alpha and two beta subunits that function as allosteric enzymes in which the alpha subunit inhibits the tyrosine kinase activity of the beta subunit. Once IGF-I or insulin is bound the receptor, autophosphorylation occurs. This provides docking sites for IRS (Insulin Receptor Substrates), which, in turn, are phosphorylated. Tyrosine-phosphorylated IRS then displays binding sites for numerous signaling partners. PI3K phosphorylates PIP2 to PIP3 (Phosphatidylinositol −3, 4, 5-Triphosphate). Akt possesses a domain that interacts directly with PIP3. Akt is required for insulin-stimulated glucose transport. GLUT4 (Glucose Transporter Protein-4) is ultimately translocated from an intracellular compartment to the plasma membrane, which results in increased glucose uptake.

IGF-I is a hormone similar in structure to insulin, produced primarily by the liver when stimulated by growth hormone. IGF-I is also universally expressed and therefore may have specific local effects. The majority (99%) of IGF-I is bound to one of seven binding proteins. IGF-I is involved in proliferation, differentiation, and glucose regulation, via the activation of signaling pathways including RAS/RAF/MEK and PI3K/AKT [6]. Although insulin and IGF-I play distinct physiological roles, they share some of the same signaling pathways. IGF-I can act independently of insulin or enhance the effects of insulin. The activation of the phosphoinositide 3-kinase (PI3K) and Akt pathway regulates metabolism. Once insulin or IGF is bound to its receptor, the receptor is autophosphorylated. Resultant phosphotyrosine motifs serve as docking sites for insulin receptor substrates (IRS). Bound IRS are then phosphorylated generating docking sites for the PI3K, which results in the conversion of PIP2 to PIP3. PIP3 recruits Akt to the plasma membrane, where it is phosphorylated [8] (Figure 2).

IGF-1R is a transmembrane receptor that is activated by IGF-I. It belongs to the large class of tyrosine kinase receptors and has a high degree of homology with the insulin receptor (IR). It is well established that IR and IGF-1R can form hybrid receptors that have a lower affinity for insulin [9] (Figure 2). Hybrid receptor abundance is increased in skeletal muscle of obese subjects and is significantly related to BMI [10]. *In vitro* studies, in a range of cell types, have suggested that reduction of IGF-1R enhances insulin sensitivity [11], [12], [13], [14], possibly by reducing hybrid receptor numbers. Therefore, manipulation of IGF-1R/IR stoichiometry may represent a novel approach to enhance insulin sensitivity and a potential treatment for insulin resistant individuals.

Low circulating IGF-I levels are associated with diabetes among adults [15] and could be partly genetically determined. In a population-based study, it was found that the absence of the wild-type (192-bp) allele of a genetic polymorphism in the regulatory region of the IGF-I gene was significantly associated with low serum levels of IGF-I. The absence of this allele was also significantly associated with an increased risk for type 2 diabetes and myocardial infarction [16], [17].

In healthy conditions, IGF-I acts in a coordinated manner with insulin to lower glycemia [18] and given to healthy volunteers increases glucose disposal [19]. When insulin resistance becomes apparent, but glucose levels are still within the normal range, there is an initial rise in circulating IGF-I bioactivity. However, when impaired fasting blood glucose develops, IGF-I bioactivity reaches a plateau. Finally, when blood glucose levels have risen and the patient is classified as having T2DM, circulating IGF-I bioactivity progressively declines [20].

The association between plasma IGF concentration and insulin sensitivity is complex. In a cohort of nondiabetic subjects with a wide range of body weight, plasma IGF-I levels were positively correlated with insulin sensitivity, and negatively with insulin secretion [21]. More recent data from a large cross-sectional study in Denmark suggest a ‘U-shaped’ association between IGF-I serum concentrations and insulin sensitivity, as both low and high IGF-I levels were related to insulin resistance, when compared to subjects with intermediate IGF-I levels [22]. The biological explanation for a ‘U-shaped relationship’ has not yet been determined. Low concentrations of IGF-I also predict the development of incident diabetes in longitudinal studies [23], [24]. However, the predictive value of IGF-I levels for incident diabetes may also follow a ‘U-shaped’ relationship and is not observed across all studies. The inconsistency may reflect the fact that IGF-I concentration alone is insufficient to reflect the complexities of metabolic regulation, and assessment may be further refined by the concentrations of other components of the IGF system, including the binding proteins (discussed further below).

IGF-I itself has little traction for therapeutic use in diabetes. Recombinant IGF-I significantly lowered blood glucose and increased insulin sensitivity in a small trial of subjects with type 2 diabetes, when administered subcutaneously for 6 weeks at supraphysiological doses (100 μg/kg twice daily); however, such treatment was associated with several adverse effects which limit translation to the clinic [25].

Several studies have investigated the association of metabolic syndrome with plasma IGF-I concentration. One study suggests that there exists a U-shaped relationship between IGF-I bioactivity and the number of components of the metabolic syndrome [20]. In contrast, other studies report an inverse relationship between total IGF-I and the metabolic syndrome [26], [27], [28], [29].

Reports of association between obesity and plasma IGF concentrations are inconsistent. Data from adolescents could also be complicated by the involvement of growth hormone in the regulation of IGF. One study found that the percentage of bioactive IGF-I was higher in obese subjects than both lean and overweight subjects [30]. This differs for another study that suggests lower IGF-I status is associated with higher fat mass [31] and that, in obese T2DM subjects, there is a decrease in IGF-I expression in subcutaneous adipose tissue, which might contribute to changes in fat differentiation capacity [32], [33]. In a study of obese adolescents, the net effect was an increase in the ratio of free to total IGF-I [34]. Differences could be attributed to the different cohorts sampled and also the different read outs of IGF-I, whether it be total, free, bound or bioactive IGF-I.

Cytokine and adipokine profiles are changed in those who are obese. Adiponectin and IGF-I concentrations in extremely obese women are positively correlated [35]; however, an inverse association between IGF-I and adiponectin is seen in Japanese men with T2DM [36]. Serum leptin concentrations are positively associated with circulating IGF components in lean elderly subjects. In contrast, no correlation was observed in moderately overweight and obese individuals [37]. In adolescents with T1DM and chronic, poor glucose control, increased serum IL-8 is associated with reduced IGF-I [38]. Obesity is associated with resistance to IGF-I at a whole-body level and in the endothelium [39]. We have previously shown that IGF-I increases eNOS phosphorylation *in vivo*, increases eNOS activity, and leads to nitric oxide dependent relaxation of conduit vessels; therefore, targeting vascular IGF-I resistance may represent a novel therapeutic target to prevent or slow the accelerated vascular disease seen in patients with obesity or T2DM [39].

### 2.2 IGF-II

IGF-II has a high degree of structural homology to IGF-I and insulin. However, compared to IGF-I, IGF-II has an increased affinity for IGF-2R. IGF-II has key roles in foetal development and its epigenetic regulation plays major roles in developmental and growth related conditions [6]. Although recent data are lacking, older investigations revealed that polymorphisms and gene variants within the IGF-II gene affect BMI and insulin sensitivity [40], [41], [42]. Epigenetic regulation, for example, DNA methylation of the IGF-II gene, may be an important determinant of childhood obesity [43]. Genetic or epigenetic variation could potentially be exploited clinically to identify individuals at higher risk of developing obesity, metabolic syndrome or diabetes in later life.

IGF-2R is identical to the mannose 6-phosphate receptor and thus binds IGF-II at the cell surface and mannose-6-phosphate tagged proteins in the *trans*-Golgi network. IGF-2R activation by IGF-II is not associated with any intracellular signaling; instead, IGF-II becomes internalized and degraded. IGF-2R is up-regulated in morbid obesity, down-regulated by weight loss and elevated in moderately obese T2DM, suggesting that IGF-2R is nutritionally regulated, independently of IGF-II [44].

### 2.3 IGFBP-1

Circulating IGFBP-1 is a 30 kDa protein, derived mainly from the liver. It is a unique member of the IGFBPs, as its circulating levels are acutely and dynamically regulated, mainly through nutritional cues [45]. Changes in insulin concentrations form the primary mechanisms by which IGFBP-1 levels are regulated via an insulin-response element in the IGFBP-1 promoter region, which confers insulin inhibition of IGFBP-1 expression [46], [47]. In the non-fed state, IGFBP-1 levels are high because of the low inhibitory effect of insulin and the stimulatory effect of cortisol and glucagon on hepatic IGFBP-1 transcription [48]. In the post-prandial state, insulin levels rise, and IGFBP-1 levels fall rapidly; the resulting increased IGF-I bioactivity augments the insulin-like actions of IGF-I. Long term calorie restriction causes an increase in serum IGFBP-1 [49].

IGFBP-1 is secreted as a phospho-protein. It is important to note that phosphorylated IGFBP-1 has a higher affinity for IGF-I than for its non-phosphorylated isoform [50], [51]. Five IGFBP-1 phosphorylation sites have been identified so far (Ser95, Ser98, Ser119, Ser169, and Ser101). Functional roles of phosphorylation sites have been investigated using mutagenesis experiments. Such studies show that phosphorylation at Ser98 and Ser169 results in mild changes in IGF-I action, and the inhibitory effects on the biological activity of IGF-I are due to IGFBP-1 phosphorylation at Ser119, which enhances affinity for IGF-I and possibly stabilises the IGF-IGFBP-1 complex [52]. Highly phosphorylated circulating levels of IGFBP-1 are closely correlated with macrovascular disease and hypertension in T2DM, whereas lesser phosphorylated IGFBP-1 isoforms are associated with glycemic control, suggesting a dual role for IGFBP-1 in the regulation of IGF actions in T2DM [53].

A strong and consistent positive correlation between circulating IGFBP-1 concentrations and insulin sensitivity has been demonstrated in diverse populations, including individuals of European and Pakistani origin [54], Asian Indians [55], healthy young men [56], adults over 65 [57], obese menopausal women [58], individuals with type 1 diabetes [59] and pre-pubertal children [60], [61]. Therefore, IGFBP-1 has been proposed as a potential marker of insulin sensitivity [62], with the change in IGFBP-1 levels over a 2 h glucose tolerance test showing promising clinical potential as biomarker [63]. There is also a negative correlation between IGFBP-1 and biomarkers of cardiovascular disease such as blood pressure, BMI, Waist:Hip ratio and fasting insulin levels [54], [60], [64].

Circulating IGFBP-1 concentration and its interaction with IGF-I were identified as important determinants of the development of glucose intolerance or diabetes in 615 individuals studied over 4.5 years [23]. More recently, a low fasting IGFBP-1 concentration was shown to be predictive of the development of abnormal glucose regulation in 355 Swedish men studied over 10 years [65], with some having up to a 40-fold increased risk of developing diabetes. Similarly, a 17 year follow up of 782 patients, showed that a low IGFBP-1 predicts the development of type 2 diabetes [66]. This trend is the same in women: a study of 240 women, over 8 years, showed that IGFBP-1 was associated with increased risk of diabetes [67]. This has also been confirmed with recent findings from a large prospective study again suggesting that IGFBP-1 levels have a strong inverse associations with risk of type 2 diabetes risk in women [68]. Interestingly, a recent follow up study reported increased serum IGFBP-1 six months after gastric bypass surgery which also reported an improvement in body weight, BMI, and waist circumference [69]. While collectively these studies provide a compelling argument that IGFBP-1 is involved in metabolic homeostasis, they do not allow any conclusions to be drawn about whether IGFBP-1 is playing a direct role in modulating insulin sensitivity. It is important to note that IGFBP-1 levels actually increase by almost a third in individuals who develop T2DM [65]. Potentially, low IGFBP-1 levels enable the development of T2DM, but as T2DM develops, IGFBP-1 concentrations rise as a consequence of hepatic insulin resistance. A recent epigenetic study of the human IGFBP-1 gene in Swedish men demonstrated that, compared with non-diabetic controls, DNA methylation levels of the IGFBP-1 gene are higher in all T2DM patients, while IGFBP-1 serum levels are lower [70]; illustrating that there could also be potential epigenetic regulation involved in IGFBP-1 levels and diabetes.

*In vivo* studies in rodents that have tried to exploit IGFBP-1 over expression have revealed inconsistent phenotypes. Transgenic mice that over-expressed rat IGFPB1 downstream of the mouse phosphoglycerate kinase promoter have fasting hyperglycemia, hyperinsulinemia and glucose intolerance, possibly due to enhanced gluconeogenesis, hepatic insulin resistance, and increased serum gluconeogenic substrate [71], [72], [73]. Others have reported no change in glucose levels in IGFBP-1 transgenic mice [74]. We have previously shown that over expression of human IGFBP-1 under its native promoter improves insulin sensitivity, promotes nitric oxide production, lowers blood pressure and protects against atherosclerosis [75]. Whereas, mice that over-expressed human IGFBP-1 under its native promoter, only exhibit fasting hyperglycemia, hyperinsulinemia and glucose intolerance in later life [76]. Interestingly, no significant differences in blood glucose and serum insulin levels were observed in IGFBP-1 knock-out mice before and after glucose challenge [77]. The differences in phenotypes could be explained by differences in promoters, background strain or transgenes [78]. Finally, modification of IGF activity by IGFBP transgenes might have important effects on foetal growth and *in utero* growth restriction [79], [80], which in turn influences adult susceptibility to diabetes.

Studies in which IGFBP-1 was administered to adult animals avoid potential confounding effects of IGFBP-1 during pre-natal development. rhIGFBP-1 injections caused insulin release, had minimal effect on blood glucose but inhibited the hypoglycemic effect of rhIGF-I in rats [81]. While another study found that, again in rat, human IGFBP-1 blocked the hypoglycemic response to intravenous IGF-I and increases blood glucose levels when administered alone [82]. Possibly, an acute increase in IGFBP-1 might block the hypoglycemic actions of IGF-I. The source of IGFBP-1 could also cause a difference in response as native IGFBP-1 is likely to be phosphorylated, whereas recombinant IGFBP-1 (dependent of the method of production) may be less phosphorylated.

It is thought that IGFBP-1 may regulate cellular actions independently of IGF/IGF receptors, possibly via an interaction of its Arg-Gly-Asp (RGD) sequence with cell surface integrins such as α5-β1 integrin. Integrin signaling can influence metabolic pathways, for example, focal adhesion kinase is activated after integrin engagement and interacts with multiple signaling intermediates, including the insulin receptor and PI3K/AKT pathway [83], [84]. This raises the possibility that interaction of integrins with RGD-containing extracellular matrix components or other RGD-containing proteins could be exploited therapeutically to target insulin sensitivity [85]. IGFBP-1 can influence cellular actions through RGD-integrin interactions, independently of IGF, in several systems, including the stimulation of healing in a dermal wound model [86] and the stimulation of cell detachment and apoptosis in breast cancer cells [87]. We have recently shown that the RGD integrin binding domain of IGFBP-1 could be a tractable therapeutic target by enhancing insulin sensitivity and increasing pancreatic insulin secretion [88]. *In vitro*, the RGD domain of IGFBP-1 enhanced insulin signaling and insulin stimulated glucose uptake in skeletal myotubes by integrin engagement and activation of focal adhesion kinase. In pancreatic islet cells, IGFBP-1 increased insulin secretion through activation of integrin-linked kinase. Both acute administration and chronic infusion of an RGD synthetic peptide to obese C57Bl/6 mice improved glucose clearance and insulin sensitivity [88]. The potential of IGFBP-1 as a translatable insulin sensitizer and secretagogue requires further evaluation.

### 2.4 IGFBP-2

IGFBP-2 is a 36 kDa protein, encoded by the IGFBP-2 gene on human chromosome 2. Unlike IGFBP-1, IGFBP-2 has no known phospho-isoforms and little is known about other post-translational modifications. However, similarly to IGFBP-1, IGFBP-2 possesses an RGD integrin-recognition motif and also a heparin-binding domain [7]. Insulin is an important negative regulator of IGFBP-2 mRNA in the liver [89]. Plasma IGFBP-2 levels correlate with insulin resistance and could be used as a biomarker of insulin sensitivity [90] and may play an important role in the pathogenesis of obesity complications in early life [91]. There is also evidence linking IGFBP-2 concentration with metabolic status in adults. Recent findings from a large prospective study in women, showed that IGFBP-2 levels have a strong inverse association with risk of T2DM [68]. In pregnancy, higher levels of IGFBP-2 are associated with a lower risk of developing gestational diabetes mellitus [92].

IGFBP-2 concentrations correlate inversely with body mass index [93] and lower adiposity [94]. This is consistent with *in vivo* data from mice over expressing IGFBP-2, which showed IGFBP-2 as a negative regulator of postnatal growth in mice, potentially by reducing the bioavailability of IGF-I [95]. We have shown that mice over expressing human IGFBP-2 have a reduced susceptibility to obesity and also improved insulin sensitivity. This was also associated with decreased leptin levels, increased glucose sensitivity and lower blood pressure compared with obese wildtype mice [96]. Over expression of IGFBP-2 by an adenovirus leading to very high concentrations of IGFBP-2 reversed diabetes in insulin-resistant ob/ob mice, diet-induced obese mice, as well as insulin-deficient streptozotocin-treated mice [97]. In another study, administration of synthetic peptides mimicking the heparin binding domains of the native protein replicated the inhibitory effects of IGFBP-2 on obesity but did not affect glucose tolerance [100]. Therefore, the effects of IGFBP-2 on obesity susceptibility and insulin sensitivity are complex and may be influenced by different domains of IGFBP-2 protein. Yau et al. recently proposed that dynamic changes in skeletal muscle IGFBP-2 expression was responsible for enhanced insulin sensitivity in response to leptin in sheep [98]. In mice, knock-down experiments suggest that IGFBP-2 is not required either for metabolic control or for the glucoregulatory action of leptin, suggesting potential species-related differences in IGFBP-2 involvement in regulating insulin sensitivity [99]. Our *in vitro* data suggest a direct effect of IGFBP-2 preventing adipogenesis as indicated by the ability of recombinant IGFBP-2 to impair 3T3-L1 differentiation [96], possibly through its heparin binding domain [100]. Recently it has been shown that IGFBP-2 enhances glucose uptake in adipocytes and that the stimulatory effect of IGFBP-2 on glucose uptake is independent of its binding to IGF-1 [101]. Collectively, these studies provide promising evidence for a potential therapeutic strategy of increasing IGFBP-2 levels to prevent obesity and diabetes.

### 2.5 IGFBP-3

IGFBP-3 is a 31 kDa protein, encoded by the IGFBP-3 gene on human chromosome 7. IGFBP-3 forms a 150 kDa ternary complex with insulin like growth factor acid-labile subunit and the IGFs. In this form, it circulates in the plasma, prolonging the half-life of IGFs and altering their interaction with cell surface receptors. The major IGF transport function is attributed to IGFBP-3, the most abundant circulating IGFBP. It carries 90–95% or more of serum IGF-I and IGF-II [102].

IGFBP-3 reduces insulin-stimulated glucose uptake, independently of IGF binding, possibly via reduced insulin-stimulated glucose transporter-4 translocation to the plasma membrane and reduced threonine phosphorylation of Akt in 3T3-L1 adipocytes [103]. IGFBP-3 induces TNF-α production in cultured adipocytes and suppresses adiponectin expression [104]. Taken together, these studies support a role for IGFBP-3 in obesity and the pathogenesis of insulin resistance. In contrast, IGFBP-3 interferes with the PPARγ-dependent processes of adipocyte differentiation and maintenance of the gene expression characteristic of mature adipocytes [105]. In human adipocytes, IGFBP-3 inhibits TNF-α-induced NF-kB activity in an IGF independent manner, restoring glucose uptake. IGFBP-3 further inhibits TNF-α, CRP and high glucose-induced NF-kβ activity in human aortic endothelial cells and subsequently suppresses monocyte adhesion; suggesting a therapeutic target for obesity-induced insulin resistance and for events occurring in the early stages of atherosclerosis [106]. IGFBP-3 also plays a role in brown adipocyte fate. Thermogenic genes were increased in brown pre-adipocytes from IGFBP-3 overexpressing mice, while initial primary growth from these pre-adipocytes from IGFBP-3 overexpressing mice was slower then wildtype mice [107].

Over expression of hIGFBP-3 in mice results in fasting hyperglycemia, impaired glucose tolerance, insulin resistance [108], [109], and reduced glucose-stimulated insulin secretion in pancreatic islets by both IGF-dependent and IGF-independent mechanisms [110], suggesting a role in the initiation and development of insulin resistance. A global genetic deletion of IGFBP-3, also leads to an increase in fasting glucose and insulin levels, but no change in insulin sensitivity [111]. High IGFBP-3 levels are associated with increased risk of T2DM in women [68]. In obese adolescents there is a decrease in total IGFBP-3 levels and increase in proteolyzed IGFBP-3 in circulation when compared to their normal counterparts [106]. These studies are supported by a recent observation seen in a large cohort of older men, which saw both high levels and low levels of IGFBP-3 being metabolically unfavorable [112]. Collectively, available data suggest that IGFBP-3 unfavorably influences insulin resistance although may have a favorable effect on adiposity.

A ratio of IGF-I/IGFBP-3 may be more useful in assessing metabolic risk [113], as a low IGF-I/IGFBP-3 ratio is associated with an approximately 3-fold increased likelihood of having the metabolic syndrome in both men and women [114]. Promisingly, a combination of rhIGF-I and rhIGFBP-3 during hyperinsulinemia directly increases peripheral glucose uptake and in the basal state, endogenous glucose production is reduced [115]. The combination of rhIGF-I and rhIGFBP-3 has also been shown to be effective at reducing fasting and daily mean glucose in patients with T2DM [116]. The combination of rhIGF-I and rhIGFBP-3 was well tolerated and clinically effective in small trials [117], [118].

### 2.6 IGFBP-4

IGFBP-4 is a 26 kDa protein encoded by igfbp-4 gene localized to chromosome 17q. IGFBP-4 is the most ‘traditional’ of the binding proteins, since it appears that its major role is to bind IGF-I and inhibit its actions. Very few studies have shown any IGF independent effects of IGFBP-4 [119]. Proteolysis of IGFBP-4 by the metalloproteinase pregnancy-associated plasma protein-A (PAPP-A), enhances IGF-I signaling by release of IGF-I near the IGF1 receptor [120]. In cultured human adipose tissue, all IGFBPs except IGFBP-1 are expressed at the mRNA level; however, IGFBP-4 is the only binding protein to be detected at high levels in the culture medium [121] and is differently expressed between adipose depots [122]. IGFBP-4 is critically involved in adipose tissue deposition and expandability, at least in part through effects on angiogenesis during adipose depot expansion [123], [124]. Genetic deletion of IGFBP-4 in mice reduced adipose tissue depots in young animals, and reduced expansion of adipose depots in a sexually dimorphic pattern in female animals on high fat feeding [122]. In another study, expression of igfbp-4 gene was negatively associated with adipose tissue angiogenesis and vascular sprouting in mice receiving high fat diet [121]. Recombinant IGFBP-4 inhibited insulin- and IGF-stimulated vascular sprouting in mouse and human adipose tissue [121]. Details of how IGFBP-4 and PAPP-A interact to regulate adipose tissue are beginning to emerge, with recent data indicating differential expression patterns of PAPP-A in mesenteric rather than subcutaneous adipose tissue [122]. Further studies are required to examine whether IGFBP-4 proteolytic cleavage is regulated in a depot-specific manner and how this influences angiogenesis and adipose expansion. High glucose levels increase IGFBP-4 proteolysis [125]; this could be a regulatory mechanism involved in preventing the loss of IGF-I hypoglycemic effects.

### 2.7 IGFBP-5

IGFBP-5 is a 28.6 kDa protein encoded by igfbp-5 gene on chromosome 2q. IGFBP-5 is the most conserved of the IGFBPs, and is present in all vertebrates [126]. IGFBP-5 concentrations are reduced in individuals with type 1 and type 2 diabetes [127], IGFBP-5 deficient mice have an increase in size, mild glucose intolerance, and increased adiposity when compared to wild type mice [128]. In individuals with diabetes, increased levels of IGFBP-5 have been shown be associated with diabetes-related complications, such as poor wound healing [129] and profibrotic effects in diabetic cardiovascular disease [130]. Single nucleotide polymorphisms affecting IGFBP-5 expression in humans are associated with changes in adiponectin concentration [131]. IGFBP5 was identified as a differentially expressed serum protein in a proteomics analysis of pregnant women, with higher concentrations found in women who developed gestational diabetes mellitus [132]. Taken together, these studies suggest that high or low levels of IGFBP-5 may be unfavorable.

### 2.8 IGFBP-6

IGFBP-6 is a 22.8 kDa protein encoded by the igfbp-6 gene on chromosome 12. IGFBP-6 is unique among the IGFBPs for its preferred IGF-II binding specificity [133]. The N-terminal and C-terminal domains of IGFBP-6 contribute high affinity IGF binding, with the C-terminal domain conferring to its IGF-II specificity [134]. Increased IGFBP-6 levels have been linked to T1DM [135]. Patients with diabetes-related complications also had significantly higher serum IGFBP-6 levels than patients without any complication [135]. Mice that over-expressed human IGFBP-6 in the brain have mild hyperleptinemia, hyperinsulinemia, insulin resistance and a decrease in UCP-1 expression in brown adipose tissue [136]. Neither study investigated whether the effects seen were causal.

### 2.9 IGFBP-7

IGFBP-7 is a 26.4 kDa protein encoded by a gene on chromosome 4q. IGFBP-7, also known as Insulin-like growth factor binding protein related protein-1 [137], has an amino acid sequence with high similarity to the other human IGFBPs [138]. IGFBP-7 (formally known as mac25) meets structural criteria as a new member of the IGFBP family and affinity cross-linking data has shown that IGFBP-7 specifically binds IGF-I and IGF-II, indicating that it is a bona fide IGFBP [138]. Compared with IGFBP-3, the affinity of IGFBP-7 for IGFs is 5–25 fold lower [138]. IGFBP-7 DNA methylation levels and IGFBP-7 serum concentrations are increased in newly diagnosed type 2 diabetic patients [139]. Serum IGFBP-7 levels are associated with insulin resistance and the risk of metabolic syndrome [140] and are also strongly related to BMI [141]. Thus, further work is required on the newest member of the IGFBP family and its potential role in diabetes.

## 3 Concluding remarks

The association between members of the IGF-axis and obesity and T2DM have been widely investigated. This has led to the proposal of certain members of the IGF-axis, particularly IGFBP-1, as potential biomarkers of insulin sensitivity and T2DM risk classification. More recently there is new evidence emerging of causality behind some of these associations. Consistently, IGFBP-2 has direct effects on the pathogenesis of obesity and has a positive therapeutic potential. Modifying IGF-I bioactivity is increasingly recognized as a therapeutic approach for several conditions. Recent discoveries highlight the IGFBP family as an attractive target for the detection, prevention, and treatment of obesity and diabetic related diseases.
